# The accuracy of digital templating in uncemented total hip arthroplasty

**DOI:** 10.1007/s00402-018-3080-0

**Published:** 2018-12-06

**Authors:** Lukas A. Holzer, Georg Scholler, Stefan Wagner, Jörg Friesenbichler, Werner Maurer-Ertl, Andreas Leithner

**Affiliations:** 1 Department of Orthopaedics and Traumatology, Medical University of Graz, Auenbruggerplatz 5, 8036 Graz, Austria; 2 AUVA Trauma Center Klagenfurt, Klagenfurt am Wörthersee, Austria; 3 Department of Traumatology, Karl Landsteiner University of Health Sciences, Krems, Austria; 4 Department of Sociology, Johannes Keppler University Linz, Linz, Austria

**Keywords:** Total hip arthroplasty, Digital templating, X-rays, Experience, Obesity

## Abstract

**Introduction:**

Preoperative planning is an essential part of total hip arthroplasty (THA). It facilitates the surgical procedure, helps to provide the correct implant size and aims at restoring biomechanical conditions. In recent times, surgeons rely more and more on digital templating techniques. Although the conversion to picture archiving and communication system had many positive effects, there are still problems that have to be taken into consideration.

**Objectives:**

The core objective was to evaluate the impact of the planners’ experience on the accuracy of predicting component size in digital preoperative templating of THA. In addition, the influence of overweight and obesity (according to WHO-criteria), patient’s sex and component design on the accuracy of preoperative planning have been analysed.

**Materials and methods:**

The retrospective study included 632 consecutive patients who had primary uncemented THA. Digital templating was done using “syngo—EndoMap” software by Siemens Medical Solutions AG. Mann–Whitney *U* test and Kruskal–Wallis test have been used for statistical analysis.

The accuracy of predicting component size has been evaluated by comparing preoperative planned sizes with implanted sizes as documented by the surgeons. The planner’s experience was tested by comparing the reliability of preoperative planning done by senior surgeons or residents. The influence of BMI on predicting component size has been tested by comparing the accuracy of digital templating between different groups of BMI according to WHO-criteria. The same procedure has been done for evaluating the impact of patient´s sex and component design.

**Results:**

The implant size was predicted exactly in 42% for the femoral and in 37% for the acetabular component. 87% of the femoral components and 78% of the acetabular cups were accurate within one size. Digital templating of femoral implant size was significantly more reliable when done by a senior surgeon. No difference was found for the acetabular component sizes. The BMI also had an impact on estimating the correct femoral implant size. In overweight patients, planning was significantly more inaccurate than normal weight people. Differences were seen in obese patients. However, these were not significant. Accuracy of acetabular components was not affected. The design of the prostheses and the patient’s sex had no influence on predicting component size.

**Conclusions:**

Inexperience and overweight are factors that correlate with inaccuracy of preoperative digital templating in femoral components, whereas acetabular components seem to be independent of these factors.

## Introduction

Preoperative planning is an essential and integral part of total hip arthroplasty (THA). It facilitates determining the correct implant size and helps restoring physiological biomechanical conditions such as leg length, centre of rotation and lateralization [1, 2]. It has been shown that component loosening due to underestimation or periprosthetic fractures due to overestimation of implant size can be avoided [3–5]. Moreover, preoperative surgical planning improves postoperative range of motion and stability, shortens the operative time and reduces wear caused by mal-positioning of the implant components [6]. Furthermore, it allows to reduce costs by decreasing the necessity of large inventories of implants [7]. In last decade, preoperative planning of THA changed as X-rays became digitalized. Accuracy and reliability have been studied in both analogue as well as digital techniques. Both techniques show similar results of accuracy [7, 8].

As indicated above digital templating poses an essential part of THA. Still, there is a variety of factors that might negatively influence its accuracy. With the introduction of picture archiving and communication system (PACS), templates with a fixed magnification factor could not be used anymore. Image size of X-rays is no longer standardised and can vary. Thus, digital images must be calibrated to scale the dimensions shown [9]. This led to difficulties in determining the correct magnification factor, especially for obese patients [6, 10–12]. Highlighting surgical results, better results and lower revision rates of orthopaedic consultants compared with residents could be shown when performing THA [13, 14]. But still, there are little data focusing the impact of the planner’s experience affecting the accuracy of predicting component size using a digital preoperative planning software [15]. Component design is expected to be important for the surgical outcome, but there are little data concerning the effect of implant design on planning accuracy [15].

Therefore, we aimed to analyse factors (planner’s experience, body mass index (BMI), sex, implant design) that might influence the accuracy of preoperative digital templating in patients who underwent THA retrospectively.

## Materials and methods

In this study, the data of 903 patients who underwent primary uncemented THA consecutively at our department between January 2012 and December 2015 were reviewed retrospectively. Female and male patients of 18–99 years of age with primary osteoarthritis of the hip were included. Exclusion criteria were the following: prior surgical interventions in the hip, cemented THA, revision surgery, intraoperative complications such as periprosthetic fractures, malalignment of the femoral stem in postoperative a.p. X-rays (defined as 5° < varus or valgus). The exclusion criteria were applied to all identified 903 patients.

The included implants were *Allofit* cup and *Alloclassic* femoral stem (both Zimmer Inc., Warsaw, IN, USA) and *Pinnacle* cup and *Corail* femoral stem (both DePuy Synthes Inc., Warsaw, IN, USA). The *Allofit* cup is a spherical cup that is flattened at the polar zone. The cup design allows press fit implantation technique. The *Alloclassic* has a tapered stem geometry and a grit-blasted surface which provide proven initial and secondary fixation. It has a rectangular cross-section that enables rotational stability. The *Pinnacle* cup is a spherical cup with a single radius. The *Corail* stem is designed to sit in the cancellous bone. It is hydroxyapatite coated and has trapezoidal-like proximal cross-section to provide rotational stability. Due to its design the choice of size might get influenced by the quality of surrounding bone stock.

### Preoperative X-ray technique

Standard preoperative digital radiographs of the hip were obtained in anterior–posterior view. The tube to film distance was 1.15 m. A metallic radio-opaque ball with a standardised diameter of 25 mm was used as a reference for determining the magnification factor. The metallic radio-opaque ball was placed next to the greater trochanter and had to be projected in total.

### Preoperative digital templating

Preoperative digital templating has been performed by either residents or consultant surgeons with the EndoMap software system (Siemens Medical Solutions AG, Erlangen, Germany). All of the surgeries were performed by consultant surgeons. The magnification factor, leg length, femoral offset and femoral neck length were determined in the anterior–posterior radiographs of the hip. Subsequently, digital templates were used for estimating correct component size in the anterior–posterior radiographs of the hip. Leg length discrepancies had to be avoided by choosing correct femoral head size.

Accuracy of preoperative planning was determined as described before by comparing the difference between planned and implanted component sizes as documented in the surgical report [8, 11, 15–18]. Perfect matches and a variance of +/− one size were considered to be adequate. Deviations of more than one size were considered inaccurate.

Furthermore, planning accuracy has been related to the planner’s experience defined by the status of consultant surgeons or residents.

### Statistical analysis

Statistical analyses have been calculated with SPSS version 20 (IBM SPSS statistics, Chicago, IL, USA). The level of significance was *p* < 0.05. Descriptive statistics were applied for sex, age and BMI. The influences of the planner's experience the component manufacturer and the patient's sex on accuracy were analysed by Mann–Whitney *U* test. The impact of BMI according to WHO criteria on planning accuracy was investigated by Kruskal–Wallis test and paired post hoc tests.

## Results

With respect to exclusion and inclusion criteria, 632 out of 903 cases were included in this study. 55% (*n* = 350) were female and 45% (*n* = 282) were male patients. The mean age was 65.7 (± 12.1) years. Demographic data of patients can be seen in Table 1.

**Table 1:** Patients’ demographic data

Age (mean)	65.7 years	± 12.1 SD
	Occurence	Percentage
Sex		
Male	282	45.0
Female	350	55.0
BMI (WHO-classification in kg/m²)		
Underweight	3	0.5
Normal weight	181	28.6
Overweight	279	44.1
Obese	169	26.7

According to WHO criteria, 0.5% (*n* = 3) were underweight, 29% (*n* = 181) were in normal range, 44% (*n* = 279) were overweight and 27% (*n* = 169) were obese.

59% (*n* = 371) of preoperative planning have been performed by consultant surgeons, 41% (*n* = 261) by residents in orthopaedic surgery.

74% (*n* = 469) of the used component designs were DePuy Synthes Inc. (Warsaw, IN, USA), whereas 26% (*n* = 163) were from Zimmer Inc. (Warsaw, IN, USA).

### General reliability

The exact stem size was predicted in 42% (*n* = 264). Further, 45% (*n* = 283) were within a range of +/− one size. Thus, 87% (*n* = 547) of the stems have been measured accurately. Data of stem planning accuracy are presented in Table 2.

**Table 2:** Planning accuracy and deviation of implants in absolute values and percentage

	Stem size		Cup size	
	Occurence	Percentage	Occurence	Percentage
Implant size				
Perfect match	264	42.0	231	37.0
+/− 1 size	283	45.0	263	42.0
+/− 2 size	67	10.6	99	15.7
+/− 3 sizes and more	18	2.8	39	6.2

	Adequate femoral planning		Adequate acetabular planning	
Planner’s experience				
Consultants	547	87.6	281	75.7
Residents	325	85.1	214	82.0
	*z* = − 2.11; *p* = 0.035		*z* = 0.64; *p* = 0.52	
BMI				
Underweight	Eliminated because of low occurence			
Normal weight	165	91.2	147	81.2
Overweight	233	73.5	216	77.4
Obese	147	87.0	130	76.9
	*z* = 6.05; *p* = 0.049		*z* = 0.20; *p* = 0.901	
Component design				
Zimmer	145	89.0	123	75.5
DePuy	402	85.3	372	79.3
	*z* = − 1.1; *p* = 0.273		*z* = − 0.23; *p* = 0.819	
Sex				
Female	312	89.1	283	80.9
Male	235	83.3	212	75.2
	*z* = 1.52; *p* = 0.13		*z* = 1.87; *p* = 0.061	

Deviation of 1 size is considered to be adequate

37% (*n* = 231) of the cups were predicted correctly, whereas further 42% (*n* = 263) were within +/− one size. Altogether, cups were estimated correctly in 78% (*n* = 494). Data of cup planning accuracy are presented in Table 2.

### Planner’s experience

Consultants predicted femoral component size correctly in 87.6% (*n* = 547) of the cases and residents in 85.1% (*n* = 325) of the cases. The difference between consultants and residents in predicting stem size within +/− one size was statistically significant (*z* = −2.11^1^, *p* = 0.035).

Regarding cup size, differences in planning accuracy were not statistically significant (*z* = 0.64^2^, *p* = 0.52).

### BMI and planning accuracy

Regarding body weight, patients were split into four groups of BMI according to the WHO criteria: underweight, normal weight, overweight and obese. Underweight persons were ignored for calculation as this group consisted of three patients. The other groups have been analysed with Kruskal–Wallis test and paired post hoc tests.

Accurate stem size was predicted in 91.2% (*n* = 165) for normal weight, in 73.5% (*n* = 233) for overweight and in 87% (*n* = 147) for obese people. According to this, a high BMI leads to an inaccurate planning of femoral component size [*H*(2) = 6.05, *p* = 0.049]. Nevertheless, paired post hoc tests only documented a statistically significant difference of normal weight patients compared with overweight (*p* = 0.043), but not with obese, as expected.

Concerning cup size, 81.2% (*n* = 147) of normal weight patients, 77.4% (*n* = 216) of overweight and 76.9% (*n* = 130) of obese have been planned adequately. Post hoc calculations showed no statistically significance. Therefore, the impact of BMI on planning accuracy of the cup could not be approved [*H*(2) = 0.20, *p* = 0.901].

### Component design

Stem components have been planned adequately in 85.3% (*n* = 402) in case of *DePuy Corail*, and in 89% (*n* = 145) in case of *Zimmer Alloclassic*. Cup size has been predicted correctly for *DePuy Pinnacle* in 79.3% (*n* = 372) and for *Zimmer Allofit* in 75.5% (*n* = 123). Mann–Whitney *U* test attested no statistically significant difference in planning accuracy regarding different implant designs (stem: *z* = − 1.1^3^, *p* = 0.273; cup: *z* = − 0.23^4^, *p* = 0.819).

### Sex and planning accuracy

Femoral components have been planned correctly in 89.1% (*n* = 312) for female patients and in 83.3% (*n* = 235) for male patients. Cup planning has been adequate in 80.9% (*n* = 283) for females and in 75.2% (*n* = 212) for males. Analysing gender differences, Mann–Whitney *U* test showed no statistical significant difference, too (stem: *z* = 1.52^5^, *p* = 0.13; cup: *z* = 1.87^6^, *p* = 0.061).

## Discussion

We retrospectively analysed the accuracy of 632 preoperative digital THA templates. The femoral components could be predicted correctly with a range of +/− one size in 87% and acetabular components in 78%. Similar results were published by Davila et al. [11] who investigated planning accuracy in 36 patients who had undergone THA. They predicted stem size in 86% and cup size in 72% correctly (+/− one size). Efe et al. [18] found that in 169 of their included patients’ hips stems were planned in 82.3% and cups in 77.5% correctly (+/− one size). The et al. studied 173 patients with THA who had preoperative plannings. 66% of their plannings were within +/− one size for uncemented stems and 52% for uncemented cups [8]. Bertz et al. [16] predicted femoral component size in 95% (+/− one size) and acetabular component size in 94% (+/− one size) in 129 patients with either cemented or hybrid THA. But it should be taken into consideration, that they also included THA with cemented fixation. Cementation might bias results of preoperative planning accuracy. With respect to this data, preoperative digital templating is a helpful tool in the preoperative management of THA. However, it has to be pointed out that our review of the literature focuses primarily on studies documenting stratified results on uncemented THA.

With respect to the present data, higher levels of experience lead to a statistically significant higher percentage of adequate preoperative planning concerning femoral components, but not for acetabular components. This corresponds to the results of Jung et al. [15]. They also showed a positive effect of higher experience on predicting stem size, but not on cup size. Carter et al. [19] documented the influence of experience on digital templating too, but they were only focusing on femoral components. They compared the results of three planners in different levels of experience and documented a positive impact of experience on planning accuracy. High level of experience led to 95% adequate planning, moderate to 88% and low experience to 82%.

BMI affects planning accuracy of femoral components, too. Nevertheless, statistically significance can only be shown for the comparison of normal weight and overweight people. Due to mispositioning of radio-opaque reference objects when performing X-rays and consequently resulting magnification errors, a negative effect on planning accuracy could be expected [17]. However, this negative influence of obesity has not been confirmed statistically. This matches with the results of Heep et al. who could not show a correlation of body shape parameters such as BMI on the magnification of a radio-opaque reference object. [20].

Furthermore, no gender-specific differences in planning accuracy could have been seen. Thus, preoperative digital templating is reliable for both, women and men. Regarding the missing deviations in calibration of X-rays between men and women, a statistically significant difference was not expected [20].

Component design of the two different implant systems that were used in our study did not take influence on predicting implant size. In the present study straight stems were used. Jung et al. found superior planning accuracy in straight stems compared to short stems [15].

One reason for the higher percentage of accurate stem plannings might be the surgical procedure. The correct position and size of the cup can be estimated during the surgery by a sufficient acetabular reaming, whereas stem size is chosen by less obvious factors such as acoustics. Therefore, it is dependent on individual subjective perception and experience. This is of special importance as no intraoperative X-rays of stem position have been made in the studied patients. Furthermore, surgeons may tend to the planned stem size with respect to the risk of fractures due to lack of macroscopically control.

When using components of DePuy, a cup size of 52 and bigger allows a femoral head size with a diameter of 36 mm. This optimises stability and prevents dislocations [21–23]. For this reason, surgeons may tend to use greater cup sizes than determined in preoperative planning.

This study has limitations as data analysis was done retrospectively. Furthermore, templating was done by either consultants or residents. In case of a prospective study, digital planning should be done by both to analyse intraobserver variability. Furthermore, consultants could be biased as they did the planning and performed the THA. In a prospective study and perfect setting templating should be performed blinded. Another point that limits our study is the lack of a check on both intraobserver and interobserver reliability.

## Conclusion

With 87% of adequately planned femoral components and 78% of adequate acetabular components, preoperative digital planning is reliable for predicting implant size in THA. Therefore, preoperative digital templating should be integrated as a routine part in the preoperative management of THA. Furthermore, higher levels of experience lead to significantly more precise predictions of stem size. BMI partly influences digital templating. Overweight is related to inaccurate preoperative planning more often. Yet, this is not valid for predicting cup size. Therefore, special attention should be paid in overweight and obese patients.

## Footnotes

1 *U*(261,371) = 44.060.

2 *U*(261, 370) = 49.628.

3 *U*(469, 163) = 36.211.

4 *U*(469, 162) = 37.562.

5 *U*(282, 350) = 52.528.

6 *U*(281,350) = 53.159.
